# Ovulation Order Mediates a Trade-Off between Pre-Hatching and Post-Hatching Viability in an Altricial Bird

**DOI:** 10.1371/journal.pone.0001785

**Published:** 2008-03-12

**Authors:** Keith W. Sockman

**Affiliations:** Department of Biology, University of North Carolina, Chapel Hill, North Carolina, United States of America; University of St. Andrews, United Kingdom

## Abstract

Simultaneously dependent siblings often compete for parentally provided resources. This competition may lead to mortality, the probability of which may be a function, in part, of the individual offspring's production order. In birds, serial ovulation followed by hatching asynchrony of simultaneous dependents leads to differences in post-hatching survival that largely depend on ovulation (laying) order. This has led to the widespread assumption that early-laid eggs are of greater value and therefore should possess different maternally manipulated characteristics than later-laid eggs. However, this perspective ignores the potential effect of laying order on pre-hatching viability, an effect which some studies suggest should offset the effect of laying order on post-hatching viability. I examined the relationship between laying order and hatching and fledging probability in wild, free-living Lincoln's sparrows (*Melospiza lincolnii*). In broods with complete hatching success, first-laid and therefore first-hatched offspring had the highest probability of fledging, and fledging probability declined with increasing laying order. However, first-laid eggs were less likely than later-laid eggs to hatch. This effect of laying order on pre-hatching viability seemed to offset that on post-hatching viability, and, consistently, maternal investment in egg size varied little if at all with respect to laying order. These results suggest that ovulation order mediates a trade-off between pre-hatching and post-hatching viability and should encourage a re-evaluation of the solitary role post-embryonic survival often plays when researchers make assumptions about the value of propagules based on the order in which they are produced.

## Introduction

Many organisms produce offspring serially, by the consecutive ovulation and spawning of each one. In some animal groups as taxonomically diverse as beetles [1], marsupials [2], and primates (including humans), parents rear serially produced post-natal siblings simultaneously, setting the stage for a competition-mediated probability of post-natal mortality that depends, in large part, on the order in which the siblings are produced [3]. Although these effects of ovulation order have important implications for how maternal investment and manipulation of offspring traits should vary across the brood, the best maternal strategy should depend on how ovulation order influences viability not just at the post-natal stage but also at the pre-natal stage.

Serial production of simultaneous dependents occurs in the vast majority of bird species and, in most, leads to the well-studied phenomenon known as hatching asynchrony [for reviews, see 4]–[6]. At the proximate level, hatching asynchrony occurs primarily because birds begin to incubate their asynchronously laid eggs before clutch completion [7], [8]. The first-laid offspring get an early start on embryonic growth and therefore hatching and post-embryonic growth, relative to those that are laid later [8]. This gives the first laid a competitive edge over their younger siblings and leads to a developmental hierarchy among nestling brood mates. Due to this competitive and developmental hierarchy, hatching asynchrony often results in the post-hatching mortality of the late laid and thus late hatched [4], even when parentally provided resources are not particularly limiting [9], [10]. The predictability and fitness implications of high intra-brood variation in post-hatching offspring mortality driven primarily by hatching order (and therefore ovulation order) should strongly influence how mothers differentially tailor investment toward, allocate resources to, and manipulate sex of simultaneously dependent sibling eggs [11]–[17]. However, as suggested above and reasoned below, maternal manipulation of egg traits should also depend on the effects of ovulation order on pre-hatching viability.

Notwithstanding controversy surrounding the ultimate bases for hatching asynchrony [5], one of the leading hypotheses for its adaptive significance is based on the susceptibility of eggs at ambient temperatures to mortality [18]. In order to protect the eggs from freezing in cold environments or from pathogens that tend to thrive at moderate temperatures [19], [20], parents should minimize the delay in elevating egg temperatures by incubation. In most species, this may mean stimulating embryonic development before clutch completion, and thus, according to this hypothesis, the nestling hierarchy is not necessarily adaptive in itself but is instead a by-product, at least in part, of incubation onset adaptively timed to reduce embryonic mortality. Hypothetically, females could initiate incubation as soon as the first egg is laid. But typically they do not, most likely due to several factors, including the possibility that energetic constraints from egg production preclude most behavior other than foraging [6], the possibility that, in cold environments, thermal and energetic constraints preclude roosting on the nest during the early laying period (Sanders, Sockman, and Hahn, unpubl. data), and the possibility that early incubation would make hatching too asynchronous [8]. Thus, females may be faced with a trade-off between maximizing the viability of early-laid offspring by initiating incubation early in laying and maximizing their own condition and the viability of late-laid offspring by initiating incubation late in laying.

If selective forces have optimized the timing of incubation onset, one would expect ovulation order to affect embryonic survival in the opposite direction that it affects post-embryonic survival [6]. For a given timing of incubation onset and a given clutch size, as ovulation order increases within a brood, pre-hatching mortality should decline due to a decline in suboptimal temperature exposure, whereas post-hatching mortality should increase due to a decline in competitive ability induced by hatching asynchrony. Additionally, some investigators have found that early-ovulated eggs are less likely to be fertile than later-ovulated sibling eggs [21]–[23]. (Because some eggs may be infertile, I use the term propagule instead of offspring when referring to both eggs and nestlings.) In either case, relative to their later-ovulated sibling propagules, early-ovulated propagules should experience lower pre-hatching viability [e.g., 23]–[26], possibly caused by sub-optimal temperature exposure or by low fertility, but should enjoy elevated post-hatching viability due to advantages in sibling competition [4].

To my knowledge, there have been no empirical tests of the hypothesis that ovulation order mediates a trade-off between pre-hatching and post-hatching viability. Support for this hypothesis would suggest that maternal manipulation of egg traits (e.g., size, steroid and anti-oxidant content, sex) should account not only for post-hatching but also for pre-hatching differences in viability with respect to ovulation order. I tested this hypothesis in a population of wild, free-living Lincoln's sparrows (*Melospiza lincolnii*). Like most bird species, Lincoln's sparrows lay one egg per day. Females begin incubating before clutch completion, and males do not incubate at all. The spring and summer breeding season occurs at high elevation or latitude [27], [28], environments that expose unincubated eggs to nighttime and early morning freezing temperatures and to daytime ambient temperatures warm enough to foster the growth of pathogenic microbes (Sockman unpubl. data). I found evidence suggesting that ovulation order may indeed mediate a trade-off between pre-hatching and post-hatching viability, evidence which should encourage a re-evaluation of the solitary role post-embryonic viability often plays when researchers make assumptions about the value of propagules based on the order in which they are produced.

## Materials and Methods

### Study site and natural history of species

This study is based on data collected during the 2005–2007 Lincoln's sparrow breeding seasons near Molas Pass, Colorado, USA. At an elevation of 3200 m, the study site (37.74°N, 107.69°W) is a sub-alpine, wet meadow ca. 20 ha in area. Like most sub-alpine habitats, this region is characterized by short summers during which nighttime lows below freezing, cold rain, brief snow squalls, and strong hail and thunderstorms are frequent. Breeding seasons for these types of sub-alpine species are often brief, harsh, and with a short-lived but strong pulse of food resources on which young are reared [29].

Adults arrive on breeding grounds in May, and, after a period of courtship by males, females initiate clutches throughout June. Individuals build open-cup nests on the ground, usually beneath a small, ca. 1/2-m high willow (*Salix glauca* and *Salix wolfii*). Clutch size varies from 3–5 eggs, and incubation lasts approximately 13 days. The asynchronously hatched nestlings are dependent on parentally provided resources for an additional 8–12 days before they fledge and gradually become independent.

### Data collection

Field assistants and I found nests by searching habitat. I estimated the date of clutch initiation for nests found during laying by subtracting the number of eggs (assuming one laid per day) minus one from the discovery date and, for nests found during incubation, by subtracting the mean incubation period (calculated from nests of known incubation period) and the number of eggs minus one from the hatching date.

We marked eggs as they were laid, enabling us to assign to many an order of laying [and hence ovulation: 30]. We measured egg length and width with calipers to estimate volume [31]. To determine the hatching order of eggs and nestlings, we visited nests typically twice daily once the predicted time of hatching approached. This frequency was a compromise between the need for precision in estimating hatching order and the need to minimize the disruption of normal nest activity and the threat of nest predation.

At hatching, we marked nestlings for identification and weighed them with a spring-loaded scale. We weighed nestlings on 2–4 additional occasions over the course of the nestling cycle, usually up through 7–8 d of age, after which we avoided handling them to prevent premature fledging. I report all ages as the age of the individual nestling, not of the brood. Frequently, hatching order of eggs was obvious based on which egg had been replaced by a new hatchling. Hatching order of nestlings was frequently obvious based either on which individual was new during a particular visit or, in the presence of multiple new hatchlings, by overt differences in dampness, which I confirmed as an indicator of hatching order by having two observers sort new nestlings according to their dampness. For this validation procedure, we used only those nestlings with a hatching order that was known but not by the observers. The observers correctly predicted hatching order 13 of 13 times. Moreover, of the 30 eggs for which I knew both laying and hatching order, 29 hatched in the order they were laid. Thus, with 97% certainty, I could infer laying order from hatching order or vice versa. I excluded from analyses eggs and nestlings for which we could not determine laying or hatching order.

### Analyses

The primary interest in this study is whether the difference between a propagule's pre-hatching and post-hatching viability depends on whether it has younger siblings, older siblings, or both (i.e., on its ovulation order relative to its siblings' ovulation orders). Given the variability of clutch size in this system, classifying ovulation order as first, second, third, etc. would not enable me to explore this primary interest, because whether egg three, for example, has only older siblings (3-egg clutches) or has both younger and older siblings (4- and 5-egg clutches) depends on clutch size. There are three analytical approaches that could potentially account for this and other confounding effects of clutch-size variation. First, I could analyze only one of the three clutch sizes. The drawbacks with this approach are twofold; I would not know whether results generalize to other clutch sizes, and this approach reduces sample size and thus the power to observe a real effect. Second, I could account for clutch size variation by including it as a covariate in statistical models. Because eggs are laid at a rate of one per day, as clutch size increases, there must be a concurrent increase in the elapsed time between clutch and incubation initiation, in the elapsed time between incubation initiation and clutch completion, or in both. Therefore, as clutch size increases, the increase in mortality should increase as well, but the magnitude of this increase should vary with ovulation order [see 32], [33]. In other words, the effect of clutch size on mortality should result from its interaction with ovulation order. Although I have enough data from each clutch size to adequately examine the role of clutch size on mortality in general, some combinations of ovulation order and clutch size are too underrepresented to adequately test this interaction. Moreover, simply including clutch size as a nuisance variable does not resolve the problem that some ovulation orders (i.e., fourth and fifth) do not occur for all clutch sizes and that some ovulation orders differ between clutch sizes in terms of the factor of primary interest—whether or not the propagule of a particular ovulation order has younger siblings, older siblings, or both. Therefore, I took the third approach, as follows. I defined an egg's laying and hatching order categorically—as first, middle, or last—and included clutch size (but not its interaction with ovulation order) as a variable. For those four-egg nests in which I had the relevant data for two middle-laid eggs (eggs two and three), I randomly selected one for the middle category. For five-egg nests, I used the third as the middle laid, unless I did not have the relevant data on it, in which case I used egg two or four, randomly selecting one when I had data for both. Thus, I used no more than three eggs or nestlings per nest, one first laid, one middle laid, and one last laid.

This approach does not enable me to test the effect of actual ovulation order (i.e., first, second, third, as opposed to first, middle, last), which also may be of interest. In other words, what is the effect on mortality of being third ovulated, regardless of clutch size and thus regardless of whether or not there are younger siblings? Determining whether a propagule is middle ovulated is more likely than determining its precise ovulation order. That is, on one visit, a nest may have one new nestling and three eggs; on the next visit, it may have the previously hatched nestling, two new nestlings of uncertain hatching order, and one egg; and on the next visit it may have four nestlings. If I did not observe the laying of these eggs, I can readily infer from hatching order whether an offspring is first, middle, or last ovulated. However, I cannot infer the second and third ovulated in this example. Consequently, conducting analyses of actual ovulation order required a reduction in sample sizes that was sometimes quite substantial, leading to lower power. Because of this and the fact that two of the three most important of these analyses (the effects of hatching order on fledging probability and of laying order on hatching and then fledging probability) failed to converge on a solution, I do not report these results.

As is typical of field studies, sample sizes varied greatly, resulting in numerous individuals or broods for which I had only partial information. For example, for any one brood, I may have known hatching order of some but not all offspring. Because of the unbalanced, hierarchical combination of fixed and random effects (e.g., measuring points nested within individuals nested within broods), each of which may differ from the others in its correlation structure, I used a mixed-model framework (Stata IC 10.0 for the Macintosh, Stata Corporation, College Station, Texas, USA) [34]–[36] to analyze how ovulation order predicts hatching probability, nestling growth, fledging probability, and egg volume. When responses were dichotomous, I used generalized estimating equations (xtgee) with robust standard errors adjusted for clustering on brood and with a logit link transformation [ln(*p*/(1−*p*))], where *p* is the probability of an outcome (hatching and fledging), to allow the probability to be bounded between 0 and 1 and to depend linearly on the predictors [37]. For continuous responses, I used multi-level mixed-effects linear regressions (xtmixed). Ovulation (laying and hatching) order, clutch size, and year (see results) were each three-level categorical variables, expanded into dummy-variable sets to model the contrast between the first and middle value of each category and the independent contrast between the middle and last value of each category (i.e., first ovulated, three-egg clutch, and 2005 contrasted with middle ovulated, four-egg clutch, and 2006, respectively; and middle ovulated, four-egg clutch, and 2006 contrasted with last ovulated, five-egg clutch, and 2007, respectively). Z-tests were conducted on the null hypothesis that a coefficient equaled 0. See Sockman et al. [17] for a detailed description of mixed, multi-level modeling frameworks, specifically as it pertains to performance of offspring clustered in broods.

## Results

Of 208 nests found over the three years of this study, 162 hatched at least one egg. Eleven of 19 three-egg nests, 40 of 74 four-egg nests, and 14 of 30 five-egg nests had 100% hatching success, although, for some of these, I did not know hatching or laying order of any of the offspring. I did not know clutch size or hatching success for the remaining nests. Among nests with 100% hatching success, the mean±95% C.I. for hatching latency (from the first-laid egg) of middle- and last-laid eggs was, respectively, 0.278±0.094 (N = 36 latencies) and 0.641±0.129 (N = 39 latencies) days.

### Effects of hatching order on fledging probability in nests with complete hatching success

**Figure 1:**
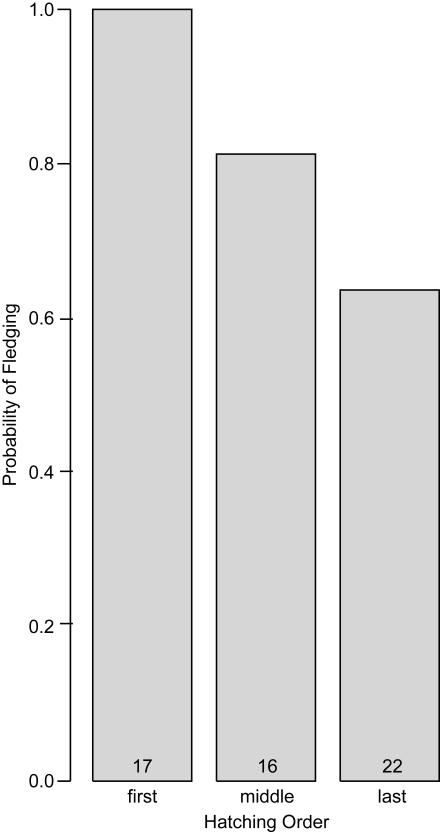
Relationship between hatching order and fledging probability in Lincoln's sparrow broods with complete hatching success. Numbers of nestlings in each category are indicated at the base of bars.

To analyze the probability of fledging in nests with complete hatching success, I nested offspring (N = 55) within brood (N = 26) and included the independent contrasts for hatching order as predictors. I also included date of clutch initiation and the independent contrasts for clutch size and year as predictors to control for these potential effects. In this particular analysis, I did not control for egg volume because adding this variable to the above model and to simpler models prevented them from converging on a solution. Hatching order had a clear negative influence on fledging probability (Table 1), revealed by the statistically reliable drop from 1.00 in first-hatched (and therefore first-ovulated and -laid) offspring to 0.81 in middle-hatched (and therefore middle-ovulated and -laid) offspring (Figure 1). Although fledging probability dropped further from the middle-hatched to 0.64 in the last-hatched (and therefore last-ovulated and -laid) offspring, this contrast was not particularly reliable from a statistical perspective (Table 1). Additionally, fledging probability increased from 2006 to 2007. I observed no clear effect of clutch size, date of clutch initiation, or the contrast between 2005 and 2006 (Table 1).

**Table 1:** Parameter estimates for modeling pre- and post-hatching viability (hatching and fledging probability, respectively), body mass of post-hatching offspring, and egg volume in Lincoln's sparrows

Response Predictor	Estimate	Standard error	z value	P value
fledging probability (55/26)				
intercept	33.443	23.753	1.41	0.159
firsthatched	7.492	3.005	2.49	0.013
lasthatched	−0.354	0.615	−0.58	>0.200
clutchsize3	−1.384	1.376	−1.01	>0.200
clutchsize5	0.757	1.379	0.55	>0.200
date	−0.209	0.146	−1.43	0.154
year2005	4.272	2.449	1.74	0.081
year2007	3.817	1.804	2.12	0.034
body mass (206/107/49)				
intercept	0.691	0.954	0.72	>0.200
age	1.016	0.162	6.27	<0.001
age^2^	0.154	0.044	3.49	<0.001
firsthatched	0.075	0.080	0.94	>0.200
lasthatched	−0.112	0.076	−1.47	0.141
age×firsthatched	−0.248	0.200	−1.24	>0.200
age×lasthatched	0.615	0.210	2.93	0.003
age^2^×firsthatched	0.073	0.548	1.34	0.181
age^2^×lasthatched	−0.228	0.063	−3.63	<0.001
clutchsize3	−0.121	0.093	−1.30	0.195
clutchsize5	−0.076	−0.086	−0.88	>0.200
date	0.007	0.006	1.16	>0.200
year2005	0.034	0.081	0.42	>0.200
year2007	0.050	0.095	0.52	>0.200
hatching probability (155/69)				
intercept	−12.300	14.988	−0.82	>0.200
firstlaid	−0.951	0.480	−1.98	0.048
lastlaid	−0.853	0.454	−1.88	0.060
clutchsize3	0.120	0.893	0.13	>0.200
clutchsize5	0.654	0.740	0.88	>0.200
date	0.059	0.085	0.69	>0.200
eggvolume	1.95	1.32	1.47	0.141
year2005	0.068	0.712	0.10	>0.200
year2007	1.208	1.502	0.80	>0.200
hatching and fledging probability (137/60)				
intercept	14.441	8.096	1.78	0.074
firstlaid	−0.388	0.367	−1.06	>0.200
lastlaid	−0.099	0.180	−0.55	>0.200
clutchsize3	0.050	1.094	0.05	>0.200
clutchsize5	0.688	0.661	1.04	>0.200
date	−0.112	0.048	−2.32	0.020
eggvolume	1.165	1.100	1.06	>0.200
year2005	2.323	0.729	3.19	0.001
year2007	2.953	1.314	2.25	0.025
egg volume (156/70)				
intercept	2.330	0.540	4.31	<0.001
firstlaid	−0.025	0.023	−1.07	>0.200
lastlaid	0.032	0.021	1.54	0.123
clutchsize3	−0.013	0.062	−0.21	>0.200
clutchsize5	−0.067	0.060	−1.12	>0.200
date	−0.001	0.003	−0.26	>0.200
year2005	0.041	0.049	0.84	>0.200
year2007	−0.032	0.064	−0.51	>0.200

The hierarchical nesting structure of each model is indicated in parentheses. Two numbers indicate the number of propagules, followed by the number of broods in which the propagules were nested. Three numbers indicate the number of measurements, followed by the number of nestlings in which measurements were nested, followed by the number of broods in which nestlings were nested. In modeling fledging probability and body mass, only broods with complete hatching success were used. In modeling body mass, only observations up through 4 days of age were used. firsthatched (firstlaid) is the contrast between first (value of 1) and middle (value of 0) hatched (laid). lasthatched (lastlaid) is the contrast between middle (value of 0) and last (value of 1) hatched (laid). clutchsize3 is the contrast between a clutch size of three eggs (value of 1) and one of four eggs (value of 0). clutchsize5 is the contrast between a clutch size of four eggs (value of 0) and one of five eggs (value of 1). year2005 is the contrast between 2005 (value of 1) and 2006 (value of 0). year2007 is the contrast between 2006 (value of 0) and 2007 (value of 1). Body mass is in g, age in days, and eggvolume in cm^3^.

### Effects of hatching order on nestling growth in nests with complete hatching success

From a proximate perspective, the negative relationship between hatching order and nestling viability has, in other studies [e.g., 29], [38], been attributed to the negative relationship between hatching order and nestling growth-rate. An analysis of growth rate is inherently biased against finding negative effects of hatching order because, among late-hatched nestlings, those that have the most robust growth rates and therefore those that are most similar to early-hatched nestlings are those that live the longest and contribute the most data to the analysis. I reduced the effects of this bias by restricting my analysis to the first half of the nestling cycle, when most of the slow growing individuals were still alive and contributing data.

**Figure 2:**
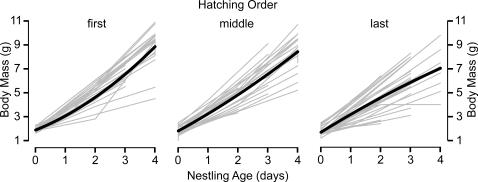
Relationship between hatching order and growth-rates in Lincoln's sparrow broods with complete hatching success. Thin, light lines are growth trajectories of individual nestlings, and thick, dark lines are population-averaged trajectories predicted from statistical models.

Not surprisingly for growth curves, a plot of body mass on age suggested a quadratic relationship (Figure 2). Therefore, using only nests with complete hatching success, I nested observation (N = 206) within offspring (N = 107) and offspring within brood (N = 49), each as a random coefficient on age and on the square of age. As predictors, I included age, the square of age, and the independent contrasts for hatching order and their interactions with age and the square of age. I also included date of clutch initiation, and the independent contrasts for clutch size and year as predictors to control for these potential effects. In this particular analysis, I did not control for egg volume because adding this variable to the above model and to simpler models prevented them from converging on a solution.

Hatching order had a clear effect on growth rate, as revealed by the effects of two interactions, that between age and the contrast of middle- and last-hatched and that between the square of age and the contrast of middle- and last-hatched (Table 1). Unlike growth rate in first- and middle-hatched nestlings, growth rate in last-hatching nestlings decreased with age (downward U-shaped curve) (Figure 2). I found no effects of clutch size, date of clutch initiation, or year on nestling mass (Table 1).

### Effects of laying order on hatching probability

**Figure 3:**
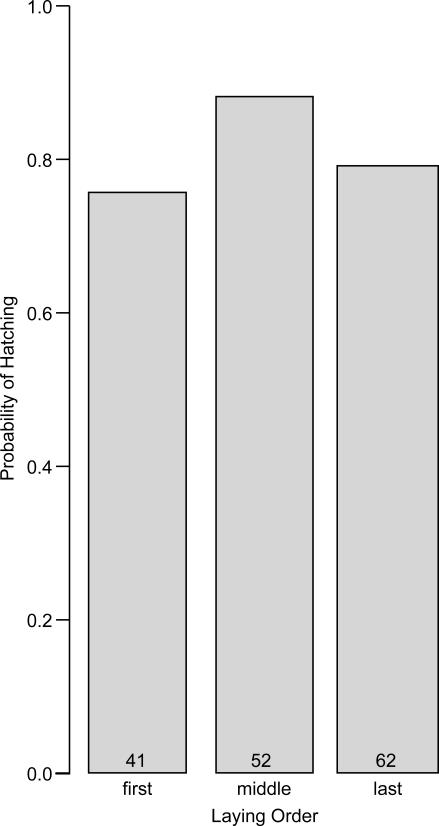
Relationship between laying order and hatching probability in Lincoln's sparrows eggs. Numbers of eggs in each category are indicated at the base of bars.

To analyze pre-hatching viability, I nested egg (N = 155) within clutch (N = 69) and included the independent contrasts for laying order as predictors. In addition, I included date of clutch initiation, egg volume, and the independent contrasts for clutch size and year as predictors to control for these potential effects. Laying order influenced pre-hatching viability, in that hatching probability of middle-laid eggs (0.88) was greater than that of first-laid eggs (0.76) (Table 1 and Figure 3). Hatching probability appeared to be lower in last-laid eggs than in middle-laid eggs (Figure 3), but this decline with laying order was not particularly reliable, nor were the effects of date of clutch initiation, egg volume, year, or clutch size (Table 1).

### Effects of laying order on hatching and fledging probability

**Figure 4:**
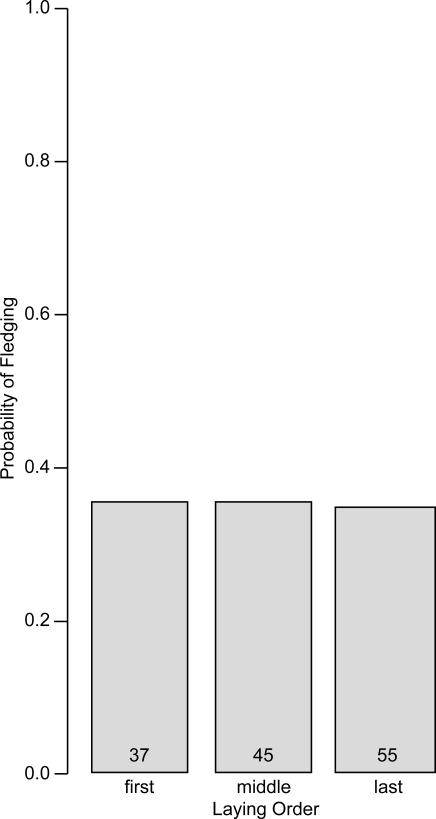
Relationship between laying order and hatching and then fledging probability in Lincoln's sparrows propagules. Numbers of propagules in each category are indicated at the base of bars.

The results so far raise the possibility that the elevation in pre-hatching viability offsets the decline in post-hatching viability with the transition from first- to middle-ovulated propagules, potentially making it no more likely that first-laid propagules would remain viable through the complete nesting cycle than later-laid propagules would. I analyzed probability to remain viable from laying through hatching and then fledging, with egg (N = 137) nested within brood (N = 60) and with the independent contrasts for laying order as predictors. I also included date of clutch initiation, egg volume, and the independent contrasts for clutch size and year as predictors to control for these potential effects. Probability of hatching and then fledging did not change with respect to laying order (Table 1) and hovered around 0.35 regardless of laying order (Figure 4). Interestingly, probability of surviving through to fledging declined with date of clutch initiation, declined from 2005 to 2006, and then increased from 2006 to 2007 (Table 1). I found no effect of clutch size or egg volume.

### Effects of laying order on egg volume

**Figure 5:**
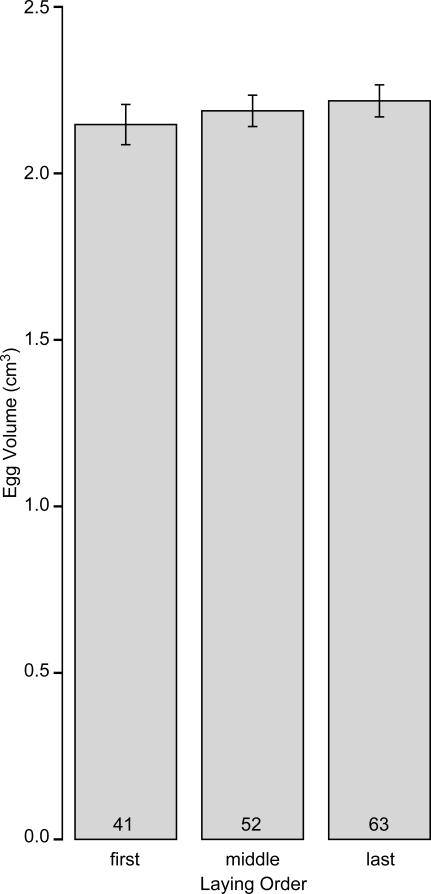
Relationship between laying order and egg volume (mean±95% C.I.) in Lincoln's sparrows. Numbers of eggs in each category are indicated at the base of bars.

Given the negligible change in hatching and then fledging probability with laying order (Figure 4), I did not expect investment to be greater in the first than in later-laid eggs, as one might expect from the results shown in Figure 1 and in numerous other studies (see Introduction). Nonetheless, I analyzed egg volume, as one component of investment, with egg (N = 156) nested within clutch (N = 70) as a random intercept and with the independent contrasts for laying order as predictors. I also included date of clutch initiation and the independent contrasts for clutch size and year as predictors to control for their potential effects. In keeping with my expectation, I observed no change in egg volume with laying order, despite fairly large samples sizes and therefore reasonable power (Table 1, Figure 5). Additionally, I found no effect of clutch size, date of clutch initiation, or year (Table 1).

## Discussion

In Lincoln's sparrows, viability during the nestling stage is highest for the first hatched of the brood and declines with later-hatched siblings. Presumably, this is due to differences in growth rates imposed by the developmental hierarchy among siblings of different age. In contrast, the first-laid propagule, which is almost always the first to hatch (if it hatches), incurs the lowest hatching rates, suggesting a trade-off between pre-hatching and post-hatching viability that is mediated by ovulation order. In other words, the maternal effect (ovulation order) that maximizes post-hatching viability is the very maternal effect that minimizes pre-hatching viability, resulting in a probability of remaining viable through the entire nest cycle that is spread relatively evenly across the serially produced, simultaneously dependent propagules. Toward that end, first-laid propagules would not seem to have the highest value, and, consistent with this, they incur no greater initial investment in terms of egg size than do later-laid propagules.

In nests of altricial species, a negative correlation between hatching order and surviving to fledge is common, if not the norm [for review, see 4]. This correlation has been attributed to hatching asynchrony, a phenomenon intensively studied for more than a half century, since David Lack [39] first provided an adaptive explanation for what was otherwise considered to be a paradox. From a proximate perspective, early hatching gives the first-laid offspring an initial competitive advantage, potentially enabling it to grow more rapidly than its younger siblings due to a positive feedback loop between competitive ability and resource acquisition. Consistent with this process, growth rate in Lincoln's sparrows negatively correlates with hatching order (Figure 2), possibly leading to the negative correlation between post-hatching survival and hatching order (Figure 1). One caveat is that the change in growth rate with respect to hatching order was most reliable from a statistical perspective for the contrast between middle- and last-hatched, even though differences in fledging probability were most reliable for the contrast between first- and middle-hatched. So, it remains to be demonstrated that the growth-rate differences actually cause the fledging-rate differences.

It is interesting to note that the mean hatching latency between first- and last-laid eggs in Lincoln's sparrows is only about 2/3 day. This may be only slightly less than the typical 1-day latencies of small, open-nesting songbirds, including another migratory Emberizid, the white-crowned sparrow (*Zonotrichia leucophrys*) [29], which is syntopic with the Lincoln's sparrow in many areas. However, it raises the interesting question regarding whether the relationship between hatching order and nestling growth and survival (Figures 1 and 2) is entirely due to hatching asynchrony or perhaps to other traits that vary with ovulation order.

Hatching asynchrony as a field of study grew, in part, out of an interest in the implications of post-natal (or post-hatching) sibling competition for an individual's inclusive fitness and consequently in how females should adjust investment in offspring and manipulate offspring or egg traits according to production order [3]. Thus, it was reasonable for investigators in this field to focus their research on broods that were complete, ignoring other periods during which viability might differ between siblings, particularly that period during the pre-natal (or pre-hatching) stage. Unfortunately, this focus fosters the specious perspective that facultative adjustment of egg traits should largely be dictated by broodmate viability-differences specifically during the post-embryonic period [e.g., 11]–[17]. In the present study, the effect of laying order on hatching probability was only marginally reliable (*P* = 0.048), raising the concern that future investigations of this relationship might yield different findings. Nonetheless, in the absence of a real effect here, it is difficult to imagine how the decline in post-hatching viability from first- to middle-ovulated propagules (Figure 1) might otherwise have been offset when examining the entire nesting period (Figure 4). Thus, in Lincoln's sparrows, it appears that the eggs producing the most robust nestlings (Figures 1) are the least likely to produce nestlings at all (Figure 3). As a consequence, the probability of remaining viable over the entire nest cycle varies little if at all among brood mates (Figure 4).

I do not know the reason first laid eggs are apparently the least likely to hatch. First laid eggs may be more susceptible to infertility than later laid eggs [e.g., 21]–[23]. Also, first laid eggs experience greater exposure to ambient temperatures than later laid eggs (Sockman unpubl. data). That this exposure raises their susceptibility to infection by pathogens or to freezing would be a reasonable hypothesis. Because the female lays, at most, one egg per day, a female that lays the modal four eggs and shows typical timing in her initiation of incubation on the laying of the penultimate egg would expose her first-laid eggs to approximately 2 days of ambient temperatures minus the duration of any brief bouts of egg warming that probably occur before incubation begins in earnest. Exposure periods such as these compromise egg viability in other avian systems [6], [18], [40], possibly because the typical ambient temperatures of most breeding environments can, during some periods of the day, foster the accumulation of various microbes, including certain types of bacteria and fungi [19], [20], [41]. In the particular breeding environment of the Lincoln's sparrow, cold is also likely to be a factor, as sub-freezing ambient temperatures are routine, even in the summer. Theoretically, the female might be able to prevent most temperature-related sources of mortality by regularly tending to the first-laid egg once it is laid. However, as suggested in the Introduction, the energetic demands of producing and laying eggs in many bird species [42], [43] and therefore the need to forage may preclude long incubation bouts during laying. Additionally, early incubation would induce more extreme hatching asynchrony, which would probably lower even further the post-hatching survival prospects of later-hatched offspring [6]. Thus, the female may be making the best of a difficult situation in balancing mortality risk of the first-ovulated with that of the later-ovulated propagules (Figure 4). From this reasoning, one might expect the last-laid eggs to be the most likely to hatch, but I have not shown this to be the case (Table 1 and Figure 3). It is possible that once the first-laid eggs of a clutch hatch, females reduce time spent incubating, so that they can feed the newly hatched nestlings [44]. This may reduce hatching probability in any egg which does not hatch early.

The lack of change in combined hatching and fledging probability with respect to laying order (Figure 4) is consistent with the almost negligible change in egg size with respect to laying order (Figure 5). Of course, adjusting egg size is only one means by which the female might manipulate offspring relative to their ovulation order. Other traits, such as the yolk's and albumen's protein, carotenoid, or steroid contents or the embryo's sex might reveal a different pattern of distribution across the laying cycle [for review, see 8]. Moreover, although I observed no relationship between egg volume and hatching probability (Table 1), it is certainly possible that other egg traits that vary with laying order influence pre- or post-hatching viability. For example, yolk androgens can vary with laying order and influence post-hatching survival or nestling growth rates in multiple species [for review, see 8]. That said, the possibility of other traits' playing a role in pre- or post-hatching propagule viability does not refute my point that investigators should exercise caution when interpreting the adaptive significance of the maternal manipulation of eggs based on post-hatching viability alone.

It would be surprising if the results I have shown here are limited to this study system or even to a very small subset of altricial species. Rather, a trade-off between pre-hatching and post-hatching viability, mediated by ovulation order, could be widespread and possibly apply to non-avian taxa with asynchronously produced propagules that are simultaneously dependent on parental care. Some researchers have examined hatching failure as a function of laying order [e.g., 21]–[26], and many have examined fledging failure as a function of hatching order [for review, see 4]. However, few, if any, have empirically shown in a single system the effect of ovulation order on hatching to be very different from the effect of ovulation order on fledging [but see 6] and then tracked viability of contemporaneous sibling propagules from ovulation through fledging. An effect of ovulation order on pre-hatching viability that offsets its effect on post-hatching viability should encourage a re-evaluation of the solitary role post-hatching viability often plays when researchers make assumptions about the value of offspring based on the order in which they are produced.
